# Acetaminophen Induced Hepatotoxicity in Wistar Rats—A Proteomic Approach

**DOI:** 10.3390/molecules21020161

**Published:** 2016-01-28

**Authors:** Soundharrajan Ilavenil, Naif Abdullah Al-Dhabi, Srisesharam Srigopalram, Young Ock Kim, Paul Agastian, Rajasekhar Baru, Ki Choon Choi, Mariadhas Valan Arasu

**Affiliations:** 1 Grassland and Forage Division, National Institute of Animal Science, RDA, Seonghwan-Eup, Cheonan-Si, Chungnam 330801, Korea; ilavenil@korea.kr (S.I.); srigopalram82@korea.kr (S.S.); 2 Department of Botany and Microbiology, Addiriyah Chair for Environmental Studies, College of Science, King Saud University, P. O. Box 2455, Riyadh 11451, Saudi Arabia; naldhabi@ksu.edu.sa; 3 Department of Medicinal Crop Research, Rural Development Administration, Eumseong, Chungbuk 369-873, Korea; kyo9128@korea.kr; 4 Research Department of Plant Biology and Biotechnology, Loyola College, Nungambakkam, Chennai, Tamil Nadu 600 034, India; agastianloyolacollege@gmail.com; 5 Proteomics Division, Discovery Research, Dr. Reddy’s Laboratories Ltd., Miyapur, Hyderabad-500049, India; barurajasekhar@gmail.com

**Keywords:** acetaminophen, MALDI-TOF, liver injury, plasma biochemical markers

## Abstract

Understanding the mechanism of chemical toxicity, which is essential for cross-species and dose extrapolations, is a major challenge for toxicologists. Standard mechanistic studies in animals for examining the toxic and pathological changes associated with the chemical exposure have often been limited to the single end point or pathways. Toxicoproteomics represents a potential aid to the toxicologist to understand the multiple pathways involved in the mechanism of toxicity and also determine the biomarkers that are possible to predictive the toxicological response. We performed an acute toxicity study in Wistar rats with the prototype liver toxin; the acetaminophen (APAP) effects on protein profiles in the liver and its correlation with the plasma biochemical markers for liver injury were analyzed. Three separate groups—control, nontoxic (150 mg/kg) and toxic dose (1500 mg/kg) of APAP—were studied. The proteins extracted from the liver were separated by 2-DE and analyzed by MALDI-TOF. The differential proteins in the gels were analyzed by BIORAD’s PDQuest software and identified by feeding the peptide mass fingerprint data to various public domain programs like Mascot and MS-Fit. The identified proteins in toxicity-induced rats were classified based on their putative protein functions, which are oxidative stress (31%), immunity (14%), neurological related (12%) and transporter proteins (2%), whereas in non-toxic dose-induced rats they were  oxidative stress (9%), immunity (6%), neurological (14%) and transporter proteins (9%). It is evident that the percentages of oxidative stress and immunity-related proteins were up-regulated in toxicity-induced rats as compared with nontoxic and control rats. Some of the liver drug metabolizing and detoxifying enzymes were depleted under toxic conditions compared with non-toxic rats. Several other proteins were identified as a first step in developing an in-house rodent liver toxicoproteomics database.

## 1. Introduction

The liver is the largest complex organ in the body which plays an important role in the internal environment maintenance by its multiple functions. It plays a central role in the metabolic pathways of carbohydrates, lipids and proteins. It is also involved in the detoxification and excretion of many endogenous and exogenous compounds by its xenobiotic metabolism. An impairment of its function is a serious health problem. Approximately 18,000 people have died per year in India due to liver function impairment [1].

Acetaminophen (*N*-acetyl-*p-*aminophenol; APAP) is widely used as an analgesic and antipyretic drug worldwide. It produces alanine derivatives by hydrolysis, which are directly converted into hydroxylamine. *N*-Acetyl-*p*-benzoquinoneimine (NAPQI), is an intermediate product of acetaminophen produced in the presence of cytochrome-p450 that causes hepatic damage [2] and tubular necrosis in the kidney [3] in both humans and experimental animals [4]. In this situation, a large amount of APAP is metabolized by the presence of P450s, which leads to reduced GSH levels by NAPQI conjugation and covalent binding of NAPQI. Acetaminophen’s clinical and biochemical side effects are well known and it is therefore used as a reference compound to assess the strengths and weaknesses of genomic and proteomic technologies as toxicological tools.

Proteomic approaches and complementary global gene expression analysis are important tools for identifying differentially expressed proteins in cells. The protein expression analysis by two dimensional electrophoresis (2DE) and matrix assisted laser desorption/ionization time of flight (MALTI-TOF) were reported for different types of diseases and offered opportunities for identifying new markers and therapeutic targets [5]. Differentially expressed proteins indicates proteins occuring in a specific function that are either the up regulated or down regulated level as compared with normal levels. The differential expression may be a consequence of disease- or disorder-related variations in transcription, translation, transport, degradation and covalent modification [6]. The systematic proteomic approach has been used to identify the proteins which are responsible for abnormal functions in the various cells. Especially, the molecular mechanisms of cell maturation [7] function [8] and pathology [9]. 2D gel electrophoresis was recently used to identify more than 1000 single proteins. The systematic proteomic approach performs different functions like energy production, protein synthesis and turnover, protein folding and transport, cell cycle, apoptosis and oxidative stress, cytoskeleton, flagella movement, signal transduction, cell recognition and metabolism as well as unknown protein functions [10,11,12]. The aim of the present study was to analyze the proteomic changes in male Wistar rat liver associated with toxicity induced by acetaminophen. 

## 2. Results

### 2.1. Biochemical Analysis

Plasma alanine amino transferase (ALT) and aspartate amino transferase (AST) levels identified by the routine clinical chemistry showed marked increases at the APAP concentration of mg/kg. The results are summarized in Table 1.

**Table 1:** AST and ALT activity of experimental group of rats.

Enzymes (IU/L)	Acetaminophen (mg/kg)		
	0 (Control)	150	1500
Aspartate aminotransferase	139 ± 4.9	115 ± 2.4	251 ± 1.2
Alanine aminotransferase	083 ± 1.5	076 ± 1.1	130 ± 8.5

**Figure 1:**
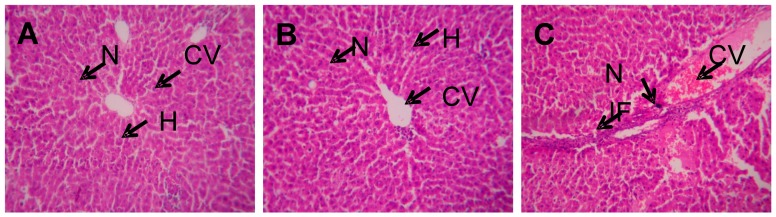
Histopathological analysis of experimental liver tissues. **A**—Control, **B**—Non toxic dose, **C**—Toxic dose induced liver tissue. N—Nucleus, H—Hepatocyte, CV—Central vein, IF—Inflammatory cells.

The control liver histopathological sections exhibited well preserved hepatocytes, nuclei, and cytoplasms with proper central veins, whereas slightly damaged hepatocytes and improper cytoplasm distribution, and infiltration of inflammatory cells around the central vein were noted in toxic dose-induced rats. Almost similar architecture was showed in non-toxic dose-induced rats (Figure 1).

### 2.2. 2D Gel Analysis

**Figure 2:**
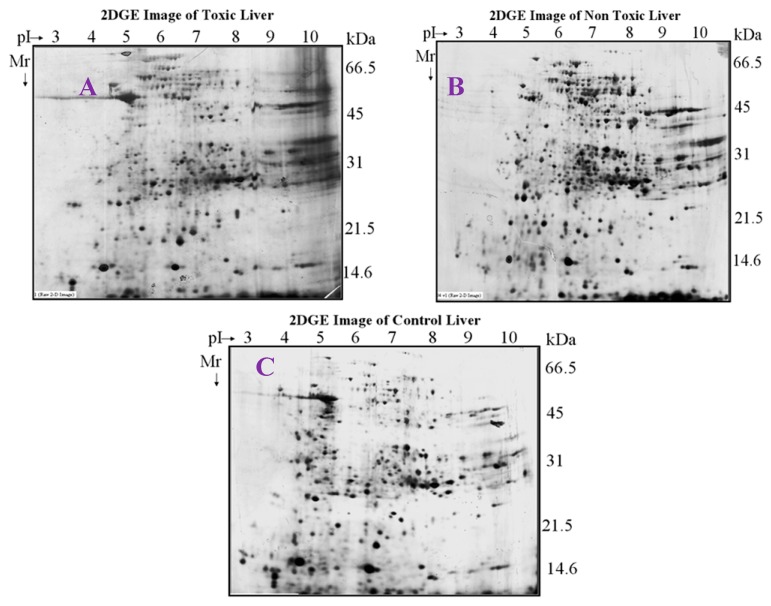
2D map of experimental rat liver. Liver proteins were separated by pH gradient 3–10 by 12% SDS-PAGE. **A**—Control, **B**—Non-toxic dose (150 mg/kg), **C**—Toxic dose (1500 mg/kg).

The proteins in the toxic, non-toxic and normal livers were separated by 2-DE on large format gels (17 × 20 cm, Figure 2). Comparison of the toxic, normal, and non-toxic dosed liver tissues protein profiles revealed significant quantitative and qualitative differences. Twenty three (23) spots were differentially expressed between control *vs.* toxic doses of the liver tissue and 39 spots were differentially expressed between the control *vs*. non-toxic doses of liver tissues, similarly, 32 spots were significantly expressed as differential protein spots between toxic and non-toxic doses.

**Figure 3:**
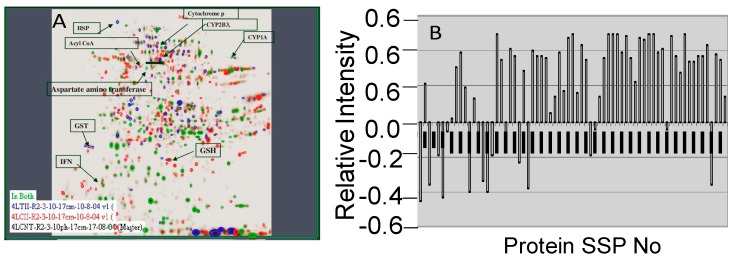
Master gel image (**A**) and relative expression of proteins (**B**).

Spots in the gel were cut and the corresponding proteins identified were indicated in Figure 3A. The relative expression levels of the proteins in experimental rats were showed in the graphical representation (Figure 3B). It showed their corresponding SSP numbers on the X axis and their relative intensities on Y-axis.

### 2.3. Image Analysis

**Figure 4:**
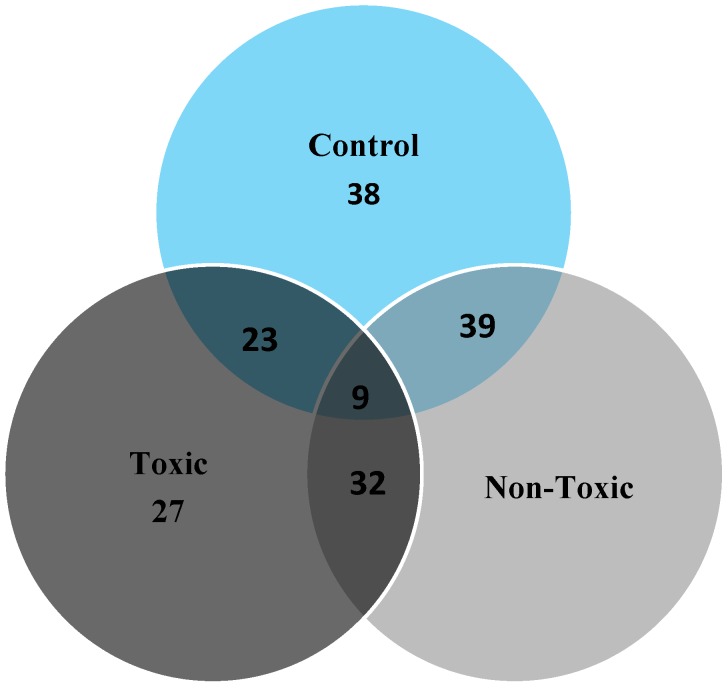
Venn graphical representation of no.of spots in the control, non-toxic and toxic dose rats.

Image analysis of the toxic, non-toxic and normal liver tissues showed number of differential proteins that were summarized in the Venn diagram (Figure 4).

### 2.4. MALDI-TOF Analysis

**Figure 5:**
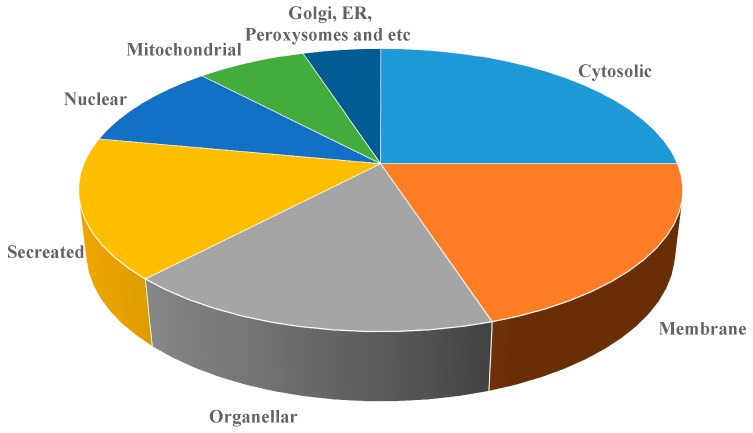
Classification of differentially expressed proteins based on the localization.

The 2D gels of toxic, non-toxic and control liver tissues were resolved on 12% SDS-PAGE and then silver stained. The selected protein spots after image analysis were taken from the gel with the listed SSP numbers and analyzed on MALDI-TOF for protein identification by submitting the peptide mass finger print data. All the protein spots were analyzed on Micromass MALDI-TOF in the reflectron mode. Based on peptide mass finger prints, the protein spots were accordingly identified by using the mascot distiller software from Matrix science UK the identified proteins are listed along with their Swissprot accession numbers (Table 2 and Table 3). The identified proteins were classified in the pie chart according to their cellular localization (Figure 5).

**Table 2:** Differentially expressed proteins in rats received 150 mg/kg of APAP.

S. No	SSP	Protein Name	Sequence Coverage (%)	Estimated M.WT	*PI*	Swissprot No
1	4514	Cold-inducible RNA-binding protein	23	38	7.0	P60825
2	3716	CYP2D3	38	57	6.7	P12938
3	5910	Tissue factor pathway inhibitor precursor (TFPI)	29	72	7.6	Q02445
4	2204	Homeobox protein DRG11	21	28.6	5.9	Q62798
5	608	SEC14-like protein 3	39			
6	3	Low-density lipoprotein receptor-related protein 4 precursor	42	45.1	4.8	Q9Z1J8
7	2308	Splice isoform 1; Variant Displayed	27	31.6	6.1	Q8VHQ7-00-00-00
8	2206	Trypsin V-B precursor	41	28	6.1	P32822
9	5	(NLG1_RAT) Splice isoform	32	13		Q62765-03-00-00
10	135	Somatostatin precursor	24	15.4	4.9	P60042
11	3402	Guanine nucleotide-binding protein G[I]	33	33	6.4	P54313
12	7602	Transforming growth factor beta 1 precursor	38	48.2	8.4	P17246
13	4801	Synaptotagmin X	47	60.7	6.8	O08625
14	7210	(EPOR_RAT) Splice isoform	42	28.2	8.9	Q07303-01-00-00
15	6412	Sphingosine 1-phosphate receptor Edg-5	39	36.6	8.2	P47752
16	3823	Hydroxymethylglutaryl-CoA synthase,	35	60.6	6.8	P17425
17	5807	Splice isoform Long; Variant Displayed;	42	61.57	7.4	Q09137-00-00-00
18	8101	Trefoil factor 2 precursor	19	15.9		Q09030
19	2808	Nonspecific lipid-transfer protein, mitochondrial precursor	34	61	6.	P11915
20	6804	Cyclic-nucleotide-gated olfactory channel	36	61.4	7.8	Q64359
21	3408	Nuclear transcription factor Y subunit gamma	29	34.4	6.5	Q62725
22	6701	Neural Wiskott-Aldrich syndrome protein	35	53.2	7.8	O08816
23	8305	Kinesin heavy chain isoform	47	29		P56536
24	2601	P2X purinoceptor 1	16	47.9	5.8	P47824
25	5108	Synaptosomal-associated protein	41	21	7.5	O70377
26	8101	Phospholipase A2 precursor	38	15.9		P04055
27	3409	3,2-trans-enoyl-CoA isomerase,	32	35.5	6.6	P23965
28	M6	Cytochrome P450	54	60.7	6.8	P30839
29	5807	Growth factor receptor-bound protein 14	36	61.5	7.4	O88900
30	6710	Methionine aminopeptidase’s 2	49	53.2	7.8	P38062
31	1206	Trypsin I, anionic precursor	51	27.2	5.5	P00762
32	1103	60 S Ribosomal protein	28	15.5	5.1	P62907
33	4101	Peripheral myelin protein 22	33	11.9	6.8	Q63199
34	M1	GST	36	24.0	4.9	P08011
35	5215	GSH	47	24	7.0	P19468

**Table 3:** Differentially expressed proteins in rats received 1500 mg/kg of APAP.

S. No	SSP	Protein Name	Sequence Coverage (%)	Score	Estimated M.WT	*PI*	Swissprot No.
1	3716	CYP2D3	25	22	57	6.7	P12938
2	8305	Homeobox protein DRG11	24	19	29	9.5	Q62798
3	3705	4-Aminobutyrate aminotransferase	35	35	56	6.4	P50554
4	3312	Serine/threonine protein phosphatase	32	34	31.5	6.7	P13353
5	6101	Acyl CoA	41	39	18.38	7.8	Q64559
6	5504	ArgininosPuccinate synthase	38	32	43.8	7.3	P09034
7	4801	Synaptotagmin X	36	38	60.7	6.8	O08625
8	8314	CD82 antigen	25	22	30		O70352
9	3823	Hydroxymethylglutaryl-CoA synthase,	29	28	60.6	6.8	P17425
10	3306	F-box/LRR-repeat protein 20	40	35	29.4	6.6	Q9QZH7
11	8301	Suppressor of cytokine signaling 1 (SOCS-1)	36	32	29.2	9.1	Q9QX78
12	1403	Calcineurin-binding protein Cabin 1	34	31	34.2	5.1	O88480
13	3115	Hydroxymethylglutaryl-CoA synthase	33	28	18.7	6.6	P22791
14	5712	Lipopolysaccharide-binding protein precursor	45	41	56	7.5	Q63313
15	3705	Cytochrome P450	46	40	56.00	6.4	P20812
16	2908	Liver carboxylesterase B-1 precursor	35	32	66.9	6.2	Q63010
17	5106	Acetyl-CoA acetyltransferase, mitochondrial precursor	33	34	20.4	7.5	P17764
18	3908	Hyaluronan synthase 2	22	17	66.4	6.6	O35776
19	3522	Carbonic anhydrase III	25	21	38.6	6.7	P14141
20	6019	Cytochrome c oxidase polypeptide VIa-liver	29	23	14.0	8.3	P10818
21	2710	CYP2B3	24	20	54.8	6.3	P13107
22	4003	Peripheral myelin protein 22	36	32	11.9	6.8	Q63199
23	8705	CYP1A2	37	35			P04799
24	M2	Heat shock protein 70	36	34	70	5.8	Q07439
25	M3	Aspartate aminotransferase	30	33	55	6.1	P13221
26	M4	Alanine aminotransferase	27	21	54		P25409
27	M5	IFN gamma	23	26	19	4.8	P01581
28	501	Mitogen-activated protein kinase	21	20	43.4	4.9	Q9WTY9
29	2710	CYP2B3	28	24	54.8	6.3	P13107
30	M6	Cytochrome P450	30	24	60.7	6.8	P30839
31	8305	Kinesin heavy chain isoform	33	32			
32	7802	(Pyruvate dehydrogenase (Lipoamide))-phosphatase 2	38	31	60	8.5	O88484
33	7602	Transforming growth factor beta 1 precursor	36	35	48.2	8.4	P17246
34	7210	Calcium-activated potassium channel beta subunit 2	41	38	28	8.9	Q811Q0
35	135	Hippocalcin-like protein	52	47	15	4.9	P62749
36	4002	Cadherin-14 (Fragment) (Rat)	17	21	11.0	6.8	Q9Z2V8
37	3	Single-stranded DNA-binding protein	20	25	14		P28042
38	850	Splice isoform Displayed	24	17	43.4	4.9	Q9R0E0
39	3512	Wnt-5a protein precursor	35	34	41.7	6.5	Q9QXQ7
40	1204	60S ribosomal protein L7	21	20	23	5.3	P37805
41	3823	Phosphoglucomutase	34	32	60.6	6.8	P38652
42	8705	Glutamate decarboxylase	36	34	60.5	9.2	P18088

**Figure 6:**
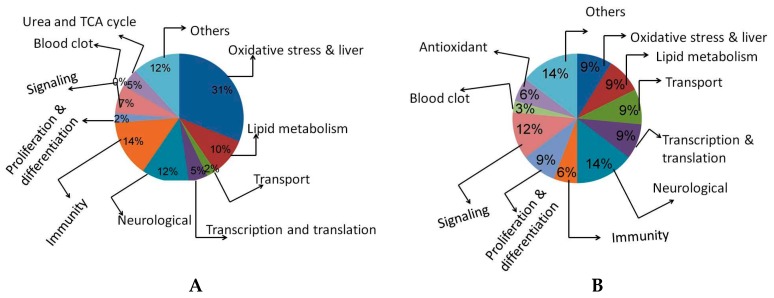
Classification of proteins based on their putative function extracted from experimental rat livers. **A**—150 mg/kg of APAP-treated rats; **B**—1500 mg/kg of APAP-treated rats.

The identified proteins were classified according to their putative functions, the percentage of transporter proteins, cell proliferation and differentiation related proteins, transcription and translational proteins, neurological and antioxidants protein were down-regulated in high dose APAP-treated rats as compared with non-toxic dose treated rats, whereas, oxidative stress, urea and TCA cycle, and immunity-related protein expressions were up regulated in higher APAP-treated rats as compared with non-toxic dose-treated rats (Figure 6).

Among the identified proteins in the toxicity induced rats, we have separated differentially expressed proteins that are known to play a vital role in the toxicity assessments, which are summarized in Table 4.

**Table 4:** High differentially expressed proteins in rats received 1500 mg/kg of APAP.

S. No	Protein Identified	Swiss Port NO	M.WT (Kda)
1	Cytochrome P450	P20812	56.00
2	Heat shock protein70	Q07439	70
3	IFN-γ	P01581	19
4	Cyp2B3	P13107	54.8
5	Cyp2D3	P12938	57
6	AST	P13221	55
7	ALT	P25409	54
8	Suppressor of cytokine signaling 1	Q9QX78	29.2
9	Liver carboxylesterase B-1 precursor	Q63010	66.9
10	Acetyl-CoA acetyltransferase, mitochondrial precursor	P17764	20.4
11	Cytochrome c oxidase polypeptide VIa-liver	P10818	14.0
12	Calcineurin-binding protein Cabin 1	O88480	34.2
13	Hydroxymethylglutaryl-CoA synthase	18.7	P22791

## 3. Discussion

The liver plays an important role in preventing the accumulation of compounds by converting them into a suitable form for elimination. All compounds undergo xenobiotic metabolism, which requires multiple biochemical transformations. During this process some of the intermediates exhibit toxic responses. Generally, the liver is potentially susceptible to injury during the action of intermediate products of compounds or drugs. An improved quantitative understanding of the balance between the xenobiotic detoxification process and hepatic injury could provide guidelines for safe levels in both pharmaceutical and the toxicological conditions. Especially, the ability to predict the toxicity profile of lead candidates is critical to streamlining pharmaceutical drug development [13]. A better understanding of the beginning of liver injury is an opportunity to recognize personalized medicine according to the genetics, active biomarkers and environment of the individual patient [14].

Acetaminophen toxicity is related to the accumulation of toxic intermediate metabolites such as *N*-acetyl-*p*-benzoquinoneimine(NAPQI). Normally, acetaminophen is metabolized into NAPQI by cytochrome P450-dependent mixed-function oxidase in the liver and by the prostaglandin synthetase system in the kidney. Then, these metabolites are detoxified by reduced glutathione (GSH). When glutathione levels are depleted, this intermediate can covalently bind to nucleophilic targets of macromolecules in cells that eventually causes cell death. In the present study, we investigated the effect of acetaminophen on liver proteomic changes in rats. The rats treated with 1500 mg/ kg of body weight exhibited increased activities of AST and ALT. This indicates that acetaminophen induced liver damage at the concentration of 1500 mg/kg. The AST and ALT levels were found higher in the cytoplasm and mitochondria. During liver damage, the transport function of hepatocytes is disturbed and as a result plasma membrane damage ocurrs thereby causing increased activities of these enzymes, further leading to cellular leakage and loss of cellular integrity [15].

The American Liver Foundation reported that 35% of severe liver failures were caused by acetaminophen toxicity. Addition of *N*-acetylcysteine to acetaminophen tablets was proposed to prevent liver toxicity [16]. The protein glutathione *S*-transferase gets up regulated during liver damage induced by excessive doses of acetaminophen. Glutathione transferases(GSTs) are complex enzymes that are involved in many biological functions, especially in detoxification of a large number of electrophilic intermediates. A large amount of electrophilic intermediates is produced by APAP oxidation in the presence of cytochrome P450. The liver is highly susceptible to these intermediates. However, these intermediates are detoxified by reduced glutathione, but higher doses of APAP exhibit more hepatotoxicity that reduce GSH levels and it permit the binding of unconjugated NAPQI to macromolecules in cells [17,18,19,20].

Many possible nucleophilic targets are found in the cells that are bound by the unconjugated NAPQI. This interaction mechanism between the cells and unconjugated NAPQI stimulates the cell death program [21,22]. Cytochrome P450s are responsible for most xenobiotics, and are needed for the proper elimination of toxic chemicals from the body. These enzymes metabolically activate biologically inert compounds into electrophilic derivatives that can cause toxicity, cell death and cancer. Advances in proteomics and genomics are providing a much improved view of the molecular players and pathways involved in the metabolism of xenobiotics. Here, we studied proteomic changes caused by treatment with higher doses of acetaminophen using 2D gel electrophoresis and MALDI-TOF. The results indicated that the percentages of transporter proteins, cell proliferation and differentiation proteins, transcription and translational proteins, neurological proteins and some of the antioxidants were down-regulated in higher APAP-treated rats as compared with non-toxic dose treated ones, whereas, oxidative stress-related proteins, urea and TCA cycle and immunity-related proteins were abundantly expressed in higher APAP-treated rats as compared with non-toxic dose treated rates.

## 4. Experimental Section

### 4.1. Animal Treatment

All animal procedures were performed under GLP conditions. Animals were separated into three groups, each consisting of five wild-type adult male Wistar rats (average weight 150–200 g) that were housed in a controlled environment and quarantined for 72 h. Group I received 0.25% CMC (control vehicle), Group II received 150 mg/kg of APAP (nontoxic dose; A5000, Sigma-Aldrich (St. Louis, MO, USA) and Group III received 1500 mg/kg of APAP (toxic dose). After the experiments the animals were sacrificed by the terminal anesthesia method and their liver tissues were removed, frozen in liquid nitrogen and stored at –80 °C.

### 4.2. Biochemical Analysis

Plasma alanine amino transferase (ALT) and aspartate amino transferase (AST) levels in the plasma toxic, non-toxic and control animals were analyzed by routine clinical chemistry.

### 4.3. Histopathology Analysis

Portion liver tissues were cut into small species and fixed in 10% formalin solution and then embedded in paraffin wax. The fixed tissues were stained with haematoxylin-eosin [12].

### 4.4. Protein Sample Preparation

Liver samples from each animal were processed separately. The whole liver was washed thoroughly in phosphate buffer saline (PBS) and finely sliced with sharp edge knife and homogenized in 5 mL of homogenization buffer I (40 mM Tris, 1 mM phenylmethylsulfonyl fluoride and 20 μL of protease inhibitor cocktail (Sigma)) on ice, with motor and pestle to a fine paste. The protein suspension was centrifuged at 15,000 rpm for 20 min at 4 °C. The pellet was dissolved in 0.5 mL buffer II (40 mM Tris, 8 M Urea, 4% CHAPS, 0.2% Biorad Biolyte [3,4,5,6,7,8,9,10], 2 mM TBP, 2 mM DTT and protease inhibitors cocktail) and centrifuged at 15,000 rpm for 20 min at 15 °C. The supernatants were aliquot and stored at −70 °C. Protein concentrations for each supernatant were estimated by Biorad’s RCDC™ method.

### 4.5. DE and Image Analysis

An equal amount of protein (300 μg) from each sample was diluted in Biorad rehydration buffer (300 µL) of and loaded onto 17 cm, pH 3–10 immobilized pH gradient IPG, strips (Biorad, Philadelphia, PA, USA). The proteins were separated on the first dimension with total of 60 kVh of Isoelectric focusing on rapid ramp using Protean IEF Cell (Biorad). The focused strips were equilibrated in buffer containing 6 M urea, 2% (*w*/*v*) SDS, 0.375 M Tris-HCl, pH 8.8, 20% (*v*/*v*) glycerol, 2.4% (*w*/*v*) acrylamide and 5 mM TBP, for 1 h at room temperature with slight agitation. The equilibrated strips were applied directly to 12.5% SDS-polyacrylamide gels (20 × 24 cm) and separated for 4 hrs at 200 V constant voltages. The gels were fixed in 40% methanol, 10% acetic acid for overnight and stained with silver stain. After rinsing, the gels were scanned with a GS800 calibrated Densitometer scanner (Biorad). Images from two replicate gels with good separation were selected for comprehensive image analysis using the PDQuest software program (Biorad, version-7). Protein profiles of samples extracted with buffer II were only considered for image analysis. Three replicate groups of the gels of control, nontoxic and toxic samples were created. Each replicate group consisted of five gels representing the liver profiles of animals in that set. The gels were normalized for any other variations like background staining, staining times, exposure times *etc.* Spots with average quantitative change greater than 2-fold between the replicate groups were considered statistically significantly regulated spots.

### 4.6. In-gel Tryptic Digestion and Mass Spectrometry (MALDI-TOF) Analysis

Spots that were significantly regulated were excised from the 2-D gel with a ProteomeWorks^TM^ spot cutter (Biorad) and placed in 96-well plates.The gel pieces were washed and destained by a mixture of 25 mM ammonium bicarbonate and 50% acetonitrile (3 × 30 min). The destained gel pieces were washed with water and reduced for 1 h at 57 °C using buffer (100 mM ammonium bicarbonate and 10 mM dithiothreitol (DTT)). The spots were then alkylated using buffer (100 mM ammonium bicarbonate and 55 mM iodoacetamide) and incubated in the dark for 30 min at room temperature. The gel pieces were dehydrated with 50 µL of 100% acetonitrile, and then dried under vacuum using the speedvac concentrator for 30 min. To the dried gel pieces, digestion solution (100 ng/µL trypsin) in 50 mM of NH_4_HCO_3_ was added and incubated overnight at 37 °C. The peptides were extracted twice from the gel pieces by adding 100 µL of extraction buffer (50% acetonitrile containing 5% trifluoroacetic acid) and the extracts were concentrated by Speedvac for 1 h. The concentrated mixture was desalted by C-18 Ziptips (Millipore, Temecula, CA, USA). The cleaned peptides crystallized with α-cyano-4-hydroxycinnamic acid (HCCA) matrix solution were analyzed by MALDI-TOF (Micromass, Temecula, CA, USA) mass spectrometry in reflectron mode. A pulsed nitrogen laser of 337 nm was fired to accumulate 100 shots per spectra and the peptide mass fingerprints (PMF) of the samples were generated. The spectra width was narrowed to a range from 500 to 3500 Daltons *m/z*. The spectra were processed (baseline correction, noise removal, deisotoping) by using the Mass Lynx version 3.5 software (Philadelphia, PA, USA). Protein identification was generated by feeding the peptide mass finger print data into the public domain search engines Mascot and MS-Fit and by searches in the Swiss Prot database (http://www.ebi.ac.uk/uniprot).

## 5. Conclusions

In this study, AST, ALT, CYP2B3, heat shock protein 70, cytochrome c oxidase, cytochrome P450, suppressor of cytokine signaling 1, liver carboxylesterase B-1 precursor, acetyl-CoA acetyltransferase, mitochondrial precursor, cytochrome c oxidase polypeptide VIa-liver, calcineurin-binding protein cabin 1, andhydroxymethylglutaryl-CoA synthasewere highly up regulated in toxic dose-treated rats. The overall resultssuggest that overdoses of drug treatments modify the regularmetabolic andmolecular pathways in the liver. These data should be useful to predict the toxicological changes in liver proteins by the overdose of acetaminophen and cholesterol lowering drugs.
